# Nitric Oxide (NO) and NO Synthases (NOS)-Based Targeted Therapy for Colon Cancer

**DOI:** 10.3390/cancers12071881

**Published:** 2020-07-13

**Authors:** Hao Wang, Liye Wang, Zuoxu Xie, Shuang Zhou, Yan Li, Yue Zhou, Meiyan Sun

**Affiliations:** 1 College of Laboratory Medicine, Jilin Medical University, Jilin 132013, China; 87115wh@163.com; 2 Department of Pharmacological and Pharmaceutical Science, College of Pharmacy, University of Houston, Houston, TX, 77204, USA; lwang53@uh.edu (L.W.); zxie3@Central.UH.EDU (Z.X.); goldenshuang1929@gmail.com (S.Z.); yli97@uh.edu (Y.L.); 3 Department of Statistics, North Dakota University, Fargo, ND 58105, USA; yue.zhou@ndsu.edu

**Keywords:** nitric oxide, NO, NO synthases, NOS, iNOS, targeted therapy, chemoprevention, NO delivery system, colon cancer, clinical manifestation, clinical implication

## Abstract

Colorectal cancer (CRC) is one of the most lethal malignancies worldwide and CRC therapy remains unsatisfactory. In recent decades, nitric oxide (NO)—a free-radical gas—plus its endogenous producer NO synthases (NOS), have attracted considerable attention. NO exerts dual effects (pro- and anti-tumor) in cancers. Endogenous levels of NO promote colon neoplasms, whereas exogenously sustained doses lead to cytotoxic functions. Importantly, NO has been implicated as an essential mediator in many signaling pathways in CRC, such as the Wnt/β-catenin and extracellular-signal-regulated kinase (ERK) pathways, which are closely associated with cancer initiation, metastasis, inflammation, and chemo-/radio-resistance. Therefore, NO/NOS have been proposed as promising targets in the regulation of CRC carcinogenesis. Clinically relevant NO-donating agents have been developed for CRC therapy to deliver a high level of NO to tumor sites. Notably, inducible NOS (iNOS) is ubiquitously over-expressed in inflammatory-associated colon cancer. The development of iNOS inhibitors contributes to targeted therapies for CRC with clinical benefits. In this review, we summarize the multifaceted mechanisms of NO-mediated networks in several hallmarks of CRC. We review the clinical manifestation and limitations of NO donors and NOS inhibitors in clinical trials. We also discuss the possible directions of NO/NOS therapies in the immediate future.

## 1. Introduction

Colorectal cancer (CRC) is the third most commonly diagnosed malignancy worldwide [1]. Epidemiology models estimate that there will be 104,610 new cases and 53,200 deaths in the U.S. among males and females in 2020 [2]. Surgery and chemotherapies are the most common strategies employed for CRC treatment. However, clinical outcomes suggest a poor five-year survival rate (<10%) in patients with advanced CRC due to distant metastasis, recurrence, chemo-/radio-resistance, and multiple drug resistance (MDR) [3,4]. Advances in understanding colon carcinogenesis, drug discovery, and drug delivery systems contribute to novel targeting strategies for CRC prevention and therapy [5].

Nitric oxide (NO) can be synthesized in mammalian cells [6]. In various tissues, this free-radical gas is endogenously generated from L-arginine by a family of enzymes named NO synthases (NOSs), including neuronal NOS (nNOS/NOS1), inducible NOS (iNOS/NOS2), and endothelial NOS (eNOS/NOS3) [7,8,9,10,11]. Among these, eNOS and nNOS are found in endothelial cells and neuronal cells, respectively. Intriguingly, eNOS and nNOS are constitutive, calcium-dependent isoforms with a low-output of NO, whereas inducible NOS (iNOS) is a calcium-independent and constantly inducible isoform. Therefore, iNOS can produce a high (nM) level of NO for an extended period. Additionally, iNOS is a macrophage-type enzyme which can be stimulated by oxidative stress, pro-inflammatory signals, and inflammatory cytokines [12,13]. NO and NOSs are involved in a plethora of physiological pathways. However, NO responses are context-dependent and highly dependent on the levels of NO. In this review, we provide an overview of the role of NO/NOSs in the hallmarks of cancer, particularly in CRC. We also discuss the conventional and innovative paradigms of NO donors and NOS chemo-inhibitors for CRC prevention and therapy.

## 2. Paradox Criteria of NO in Cancers

NO has a biphasic effect on cancer development [14]. The response to the action of NO is concentration-dependent [15]. Low levels of NO are prone to favor cell growth and anti-apoptotic responses, whereas high levels of NO are likely to induce cell cycle arrest and apoptosis [6,16]. Experiments have shown that particular signaling pathways are activated/inhibited when endothelial cells are exposed to different concentrations of NO [17,18,19,20]. Cyclic guanosine monophosphate (cGMP) signaling can be activated at the lowest doses of NO, inducing the proliferation of human endothelial cancer cells. The cGMP pathway acts as a critical player, mediating either the acute response to NO (5 min to 1 h) or the long-term proliferation response [21]. As NO doses increase, PI3 kinase-Akt signaling can be directly activated by NO, which is associated with migration and angiogenesis in endothelial cells [22]. However, at higher local concentrations (>1 µm), NO stabilizes the hypoxia-inducible factors (HIF-1α), which is linked with suppressed cell proliferation and delayed wound repair [23]. Moreover, when the cells are exposed to a substantial amount of NO, the growth will be hindered due to the accumulation of p53 [24]. Indeed, accumulated p53 is found in human cells when exposed to high levels of NO, which is produced by a NO donor or by the overexpression of iNOS. An increase in iNOS expression and p53 mutations are also found at the inflamed colon sites of patients with ulcerative colitis (UC) [25].

Consistently, studies have reported that, in breast cancer cells (MCF7 cells), signaling proteins respond to various amounts of NO. Extracellular-signal-regulated kinase (ERK) is phosphorylated at the NO level of 10–30 nM, and Akt is phosphorylated at 30–60 nM NO. When NO reaches 100 nM, HIF-1α is stabilized. Around 400 nM, such a high output of NO initiates the phosphorylation of p53 [15].

Interestingly, a previous study has indicated that NO supports tumor growth and mediates tumor vascularization at an early stage but may oppose such actions at advanced stages with metastasis. This is consistent with the finding that the expression of iNOS reduces with the advance of tumor stages and is low or undetectable in metastasis to the lung and liver [26,27].

## 3. Biphasic Role of NO in Cancers

In the role of NO in cancer tumorigenesis, the low levels of NO generated by NOS lead to tumor initiation and progression by several mechanisms: (ⅰ) NO induces DNA damage; (ⅱ) NO interferes with DNA repair; (ⅲ) NO activates oncogenes; (ⅳ) NO decreases apoptosis; (ⅴ) NO causes post-translational modification; and (ⅵ) NO causes gene mutations under chronic and malignant conditions, such as the accumulation of mutant p53. Besides, NO participates in cancer-associated signaling pathways, including Wnt, Ras, ERK, Akt, cyclin D1, mammalian target of rapamycin (mTOR), and retinoblastoma (Rb) pathways. NO also mediates the events of angiogenesis, epithelial–mesenchymal transition (EMT), and metastasis [15,28,29,30,31,32,33]. It has been reported that NO increases Matrix Metallopeptidase-2 and -9 (MMP-2)/MMP-9 and activated ERK-1/2 and activating protein 1 (AP-1) in colon adenocarcinoma cells in a time-dependent manner [34].

NO exogenously provided by NO donors participates in cytotoxicity effects against colorectal cancer with the following mechanisms: (ⅰ) NO inhibits DNA synthesis; (ⅱ) NO inhibits angiogenesis; (ⅲ) NO inhibits EMT; (ⅳ) NO reduces mutations of p53; (ⅴ) NO deactivates Cyclooxygenase-2 (COX-2); (ⅵ) NO activates caspase family proteases; (ⅶ) NO alters the expression of apoptosis-associated proteins, such as the Bcl-2 family; and (ⅷ) NO decreases p21. One study revealed that NO was involved in CRC carcinogenesis in a p53-dependent manner. This demonstrated that p53-dependent miRNAs were associated with the resistance of CRC cells to NO-induced apoptosis. The upregulation of these miRNAs sensitized the NO-induced apoptotic death in HT-29 cells [35].

In recognition of the anti-tumor effects of NO, NO-donating agents, and delivery systems are being developed to manipulate NO levels in the tumor environment [12,36,37,38,39,40,41].

## 4. Role of NOS in Colon Carcinogenesis

### 4.1. nNOS/NOS1

Previous studies have demonstrated that NOS1 promotes the survival of nasopharyngeal carcinoma cells and cancer-associated fibroblasts, but little has been related to colon cancer [42,43]. A recent study suggested that mitochondrial NOS1 (mtNOS1) is involved in chemotherapeutic resistance in colon cancer cells via inducing SIRT3 activity. This finding also indicates that NOS1-induced apoptotic resistance in CRC can be overcame by inhibitors targeting NOS1 [44].

### 4.2. iNOS/NOS2

In the last few decades, iNOS has been at the center of attention in terms of its role in human colonic tumorigenesis. It has been reported that iNOS is highly expressed in almost 60% of human colon adenomas, whereas it is low or undetectable in normal intestinal tissues [27,45]. Interestingly, the expression level of iNOS attenuated in an advanced tumor with metastasis, and was absent in human mesenchymal CRC subtypes [46].

Epidemiologic research has demonstrated that inflammatory bowel disease is closely correlated with CRC [47]. iNOS was frequently detected at inflammatory sites, both in patients with ulcerative colitis (UC) and colonic neoplasms. This suggested that iNOS-induced NO levels might be higher in inflammatory and tumor tissues than in normal tissues [48,49]. Indeed, higher expression levels of iNOS were found in both human colitis and carcinoma tissues than in non-carcinoma tissues [50,51,52,53]. The presence of a high iNOS level has been found in human CRC cell HT-29 under an inflammatory condition [54]. Besides, it is well-known that MMP-2 plays a critical role in invasion and metastasis, and is more active in colonic colitis and cancer compared to controls [26]. Studies have shown an increase of iNOS in MMP-2-related invasion and metastasis in colorectal cancer [11,55]. Furthermore, iNOS is involved in the NF-kB pathway [29]. These findings suggest that iNOS might be an indicator of human colon cancer and inflammation at early stages. It is known that iNOS and COX-2 are correlated with a poor prognosis and high biological aggressiveness of tumor cells [56]. One study reported an upregulation of iNOS and COX-2 in colorectal cancer [26]. The product of NO is higher in more advanced disease, thus demonstrating an essential role of iNOS in the development of CRC [9,31].

### 4.3. eNOS/NOS3

As reported in previous studies, the role of eNOS in tumorigenesis is mainly due to its aberrant expression in endothelial cells and tumors [57,58]. Indeed, a high expression of eNOS was detected in undifferentiated tumor tissues compared to differentiated tumor tissues in a murine mammary mice model [59]. An upregulation of eNOS was found in rat colon tumors induced by azoxymethane [60]. Recent studies revealed that eNOS might be involved in various tumor hallmarks, including invasion, metastasis, angiogenesis, and resistance [61,62]. Therefore, eNOS could act as a marker for poor prognosis in human cancer [46,63]. Peñarando et al. explored the mechanism of eNOS in mesenchymal colorectal cancer and cancer stem cells. They depicted that eNOS was upregulated in human colorectal tumor subtypes, including the human mesenchymal CRC subtype. In patients with CRC, eNOS was also enhanced at the initial stage, along with *APC* loss [60]. Notably, the authors demonstrated that eNOS was highly expressed in different cancer stem cell (CSC) phenotypes, which included different conditional CRC mice models, poorly differentiated adenocarcinomas, and human mesenchymal CMS tumors. This finding identified eNOS as a possible novel biomarker in poor-prognostic mesenchymal colorectal tumors. Moreover, a new NO scavenger named cPTIO was found, which impaired the stem-related signaling pathways in the CSC phenotypes, and inhibited organoid and tumor formation. Their study suggested eNOS as a promising target in human mesenchymal colorectal tumors [46].

## 5. NOS Inhibitors: Targeting NOS in Colon Cancer

As mentioned above, all of the NOS isoforms play an essential role in the development of colon cancer. Therefore, in the past few years, various NOS inhibitors have been developed [28]. NOS inhibitors have been preclinically determined to reduce the endogenous production of NO, and thus suppress colonic tumor and inflammatory formation [30]. However, due to the constitutive expression and central role of eNOS in smooth muscle relaxation and the control of vascular tone and blood pressure, the inhibition of eNOS may result in unexpected side effects. Moreover, several studies have shown an increase in iNOS expression in human colon adenomas [31]. Therefore, scientists have been making efforts to find selective iNOS inhibitors. In addition, some researchers believe that iNOS-specific inhibitors could be developed as safer and more effective chemo-preventive agents against colon cancer in comparison to COX-2 inhibitors, which may cause renal toxicity [26]. Here, we discuss typical examples of NOS inhibitors, which are classified into two categories: natural extracts and synthesized compounds. The key information—including the targets, mechanism, and inhibited protein—are listed in Table 1.

### 5.1. NOS Inhibitors from Natural Extracts

#### 5.1.1. Celastrol (Tripterine)

Our recent study [66] demonstrated that celastrol (tripterine), extracted from the thunder god vine, inhibited the activities of iNOS and eNOS in CRC cells. The inhibitory effects in cell growth and migration were associated with the angiogenesis pathway. This was the first study on the combinational use of the NOS inhibitor celastrol with first-line chemotherapy reagents, such as 5-FU, in CRC. It showed synergistic anti-proliferation effects in CRC cells.

#### 5.1.2. Maqui Berry (MB) Extracts

It has been reported that Maqui berry (MB) extracts downregulated the expression of iNOS, resulting in the inhibition of NO production in human CRC cells. This study provided evidence that MB extracts potently protect against CRC. Moreover, the extracts showed antioxidant and anti-inflammatory abilities in human CRC cells, as the structural feature of extracts might be associated with the inhibition of NF-κB and COX-2 [67].

#### 5.1.3. Cannabidiol (CBD)

Jeong et al. first reported that the combined administration of CBD and oxaliplatin not only inhibited tumor growth, but also induced autophagic death by decreasing phospho-eNOS and SOD2 in oxaliplatin-resistant CRC cells, which suggested that CBD could work as a new therapeutic agent to overcome the chemo-resistance of oxaliplatin in human CRC cells, sparing the neurotoxic side effects of oxaliplatin [77].

#### 5.1.4. Phaleria Macrocarpa

A recent study determined the inhibitory effects of an ethanol extract, *Phaleria macrocarpa* stem bark, on the expression of iNOS in CRC cell line HCT116 [76].

#### 5.1.5. All-Trans Retinoic Acid (AtRA)

Rafa et al. suggested that AtRA exerted a clinically preventive effect in patients with ulcerative colitis (UC) and colitis-associated cancer (CAC). Mechanically, AtRA regulated the TLR-4/NF-κ;B pathway targeting iNOS in colonic mucosa. Moreover, they revealed a correlation with the expression of iNOS and TNF-a in the colonic mucosa. AtRA inhibited the expression of iNOS and TNF-a [70]. This study offered a new strategy in which AtRA could protect against CAC and UC development.

#### 5.1.6. Dietary Polyphenol Ellagic Acid

Umesalma et al. reported that ellagic acid suppressed colon cancer in rats. In their subsequent study, they explored the precise mechanism of ellagic acid against colonic inflammation through the NF-κB pathway to reduce the expression of iNOS, TNF-a, and IL-6. Ellagic acid could be regarded as a promising chemo-preventive agent due to its anti-tumor and anti-inflammatory effects [75].

### 5.2. Synthesized NOS Inhibitors

#### 5.2.1. 1400 W and L-NIO

Our recent study [64] confirmed that the blockage of iNOS or eNOS significantly inhibited CRC cell proliferation due to the reduced level of NO. Two NOS inhibitors, 1400 W and L-NIO, hindered the CRC cell growth and migration. Additionally, we demonstrated that such an inhibitory effect of 1400 W and L-NIO on CRC cells functions, in part, by suppressing angiogenesis pathway and angiogenesis-related proteins. In addition, this was the first attempt examining the combinational use of 1400 W or L-NIO with the chemotherapy drug 5-FU, and successfully presented a synergistic anti-proliferation effect in CRC cells.

#### 5.2.2. Se,Se’-1,4-Phenylenebis(1,2-Ethanediyl)Bis-Isoselenourea (PBISe)

PBISe, a newly synthesized iNOS inhibitor, has been suggested as a potent agent for attenuating CRC cell proliferation, but inducing apoptosis. It significantly inhibited iNOS, PI3 kinase, and pAkt signaling in CRC cells. Notably, this study elucidated that upon exposure to PBISe, a decrease in the levels of pAkt and Akt2 and an increase in p27 were found in CRC cells [71]. Taken together, PBISe could be considered a novel selenium compound inhibiting CRC cell growth, while its inhibitory effects in animal models are currently under investigation.

#### 5.2.3. S,S9-1,4-Phenylene-Bis(1,2-Ethanediyl)Bis-Isothiourea (PBIT)/L-N6-(1-Iminoethyl)Lysinetetrazole-Amide (SC-51)

PBIT is an iNOS inhibitor. It has been reported that PBIT modulated iNOS and COX activities against aberrant crypt foci (ACF) in the colon of rats and colonic mucosa. Interestingly, PBIT selectively and competitively inhibited the levels of iNOS compared with eNOS or nNOS. It provided data showing that an NOS-specific inhibitor might be safer than COX-2 inhibitors, which may cause renal toxicity [26].

Another study found that the selective iNOS inhibitors L-N6-(1-iminoethyl)lysine tetrazole-amide (SC-51) and aminoguanidine (AG) selectively suppressed iNOS against colonic ACF formation. Notably, the co-administration of celecoxib, a COX-2 inhibitor, and SC-51 or AG exerted a more potent inhibition on iNOS against colonic ACF formation than when the agents were administrated individually [68]. This finding demonstrated that the combinational use of iNOS inhibitors and COX-2 inhibitors might have additional potential in CRC chemo-prevention.

### 5.3. NOS Inhibitors from Nutraceuticals

#### 5.3.1. Nobiletin and Its Colonic Metabolites (NOB-Met)

Wu et al. orally administrated NOB and its major metabolites as a mixture (NOB-Met) to CRC cells. They found that NOB-Met inhibited iNOS expression in colitis-associated colon cancer. Moreover, NOB-Met inhibited cell proliferation by increasing p27 and p53 and decreasing cyclin D, CDK2, CDK4, and CDK6 in CRC cells. This was the first study demonstrated that NOB-Met exerts anti-tumor and anti-inflammation effects in CRC via downregulating iNOS and upregulating antioxidative enzymes [69]. This study revealed that NOB-Met could be a good nutraceutical candidate for colonic cancer and inflammation prevention [69].

#### 5.3.2. Omega-3 Fatty Acid Docosahexaenoic Acid (DHA)

A previous study reported that DHA could induce CRC cell apoptosis by targeting iNOS and NF-κB. This was the first study showing that DHA modulates CRC cell growth via the inhibition of iNOS and COX-2 [74].

## 6. Traditional and Innovative NO Donor-Based Therapy

NO donor-based therapy is a newly developed strategy for cancer therapy as it (1) achieves more potent anti-tumor effects; (2) overcomes chemo-/radio-resistance; and (3) reduces gastrointestinal toxicity via an enhanced specificity. NO donor-based therapy has been advanced from an NO donor alone towards the (1) co-administration of NO donors with other chemo-/radio-/immune-therapy; (2) hybridization of NO donors with gaseous transmitters, such as hydrogen sulfide (H_2_S) and carbon monoxide (CO); (3) combinational use of iNOS and COX inhibitors; and (4) application of drug delivery systems, such as nanotechnology, to deliver a high level of NO to the tumor sites. In this section, we review the advances of NO donor-based therapy in CRC made in the past decades and provide prospects [79,80,81,82,83].

### 6.1. Nitric-Oxide-Donating Nonsteroidal Anti-Inflammatory Drugs (NO-NSAIDs) in Cancers

In 1973, Lawrence Levine first suggested that NSAIDs had an anti-tumor effect in fibrosarcoma-bearing mice [84]. In the 1980s, considerable data generated by preclinical studies, clinical investigations, and epidemiological work revealed that NSAIDs could prevent colon cancer [85,86,87,88,89,90]. Notably, a clinical study conducted by Waddell and Gerner in patients with familial adenomatous polyposis (FAP), a condition in which a person develops numerous adenomas in the colon and rectum, suggested that NSAIDs may offer a bright clinical insight into CRC protection [91]. However, due to the gastrointestinal (GI) toxicity and risk associated with NSAIDs, celecoxib, the NSAID used for FAP in clinics was withdrawn by the FDA [92,93]. Safer alternatives need to be developed [87]. NO-donating NSAIDs (NO-NSAIDs) possess a traditional NSAID moiety and an NO-donating moiety, which are covalently coupled by a spacer molecule [94]. NO–NSAIDs share the anti-cancer and pharmacological properties with their parent NSAIDs [95]. Impressively, the application of an NO-donating moiety to an NSAID might carry NO to tumor sites and compensate for the reduction of gastric prostaglandin (PG), largely contributing to the diminished gastric toxicity and mucosal injury [87,96]. To the best of our knowledge, there is no safer alternative for patients with FAP. We believe that NO-NASIDs might be a promising candidate. Moreover, we believe that the development of NO-NASIDs could rekindle researchers’ interests in FAP and CRC therapy. Herein, we list some in vitro, in vivo preclinical, and clinical examples of NO donors with different chemical properties for colon cancer therapy (Table 2 and Table 3).

### 6.2. NO-NSAIDs in CRC

Current data indicates that NO-NSAIDs exert anti-neoplasia effects on colon adenocarcinomas [95]. As has been reported, the anti-tumor effects of NO-NSAIDs are a hundred- to thousand-fold more potent than those of parent NSAIDs [94]. In 1998, Bak et al. first determined that an NO-donating aspirin (NO-ASA) derivative, NCX-4016, exhibited over a 30% reduction of aberrant crypt foci (ACF) compared to that of aspirin (NO-ASA: 85% reduction of ACF; aspirin: 64% reduction of ACF) in a rat model bearing tumors. The result supported the superior chemo-preventive effects of NO-ASA in colon adenocarcinoma [97]. Consistently, Williams et al. found that NO-NSAIDs much more potently inhibited the growth of human colon cancer cell HT-29 than the parental NSAID alone. The IC50 of NO-ASA in HT-29 cells was 10 mM within 72 h, whereas that of aspirin was 2500 mM. Ample evidence has demonstrated that NO–ASA is the most potent NO–NSAID in various cancer cell lines [104,105,106,107,108,109]. Besides, NO-NSAIDs have been regarded as safe agents in clinical studies [110,111,112,113,114,115,116,117,118,119,120,121]. Evidence also suggests that the phenotypes of NO-NSAIDs—such as NO-ASA, NO-sulindac, and NO-ibuprofen—inhibit human CRC cell proliferation more effectively than parent NO-NSAIDs [122]. NO-ASA has been proven to reach a superior effectiveness, even hundreds- or thousands-fold greater than the other NO-NSAIDs, offering a novel and effective strategy for colon cancer chemo-prevention. Mechanically, research has shown that some signaling pathways are involved in the suppressive effects of NO-ASA in colon carcinogenesis, including the Wnt and NF-kB signaling pathways, NOS, and cyclooxygenase (COX) enzymes [123,124]. For example, NO-ASA hampered the activation of NF-kB by interfering with its translocation into the nucleus [125]. NO-ASA inhibits iNOS expression in CRC cell line HT-29 [126]. Additionally, a reduction of iNOS activities has also been found in NO-ASA-treated F344 rats with azoxymethane-induced colon cancer [127]. Furthermore, a novel NO-NSAID, GT-094, inhibited CRC cell growth, in part, by activating an ROS-miR-27a:ZBTB10-Sp pathway in RKO and SW480 cells [103]. Interestingly, NO-ASA induced COX-2 expression in HT-29 and DLD-1 CRC cell lines, whereas PGE2 was inhibited under the co-administration of NO-ASA and ASA (200 µm NO-ASA; 20 µm ASA) [126]. Structurally, three positional isomers of NO-ASA—namely ortho, meta, and para, inhibited β-catenin and TCF4 signaling (1–4% of the IC50 for cell growth inhibition)—suggesting the potential of NO-ASA to interfere in prominent signaling pathways in chemoprevention for CRC or other cancers [127].

### 6.3. NO- and H_2_S-Releasing NSAIDs in CRC

NOSH-aspirin (NBS-1120, NOSH-ASA) is a hybrid incorporating two gaseous mediators: NO and H_2_S. A preclinical study showed that NOSH-aspirin inhibited tumor growth in a human colon cancer xenograft mice model [128]. Chattopadhyay et al. first developed this NO- and H_2_S-releasing NSAID and tested its efficacy on the suppression of colon cancer in vitro and in vivo [98]. Surprisingly, NOSH-aspirin was 9000-fold more potent towards CRC growth inhibition than the sum of its parts, with IC50 in the nano-molar range. Subsequent experiments confirmed the high potency of NOSH-aspirin in growth suppression, apoptosis induction, and cell cycle blockage in CRC cell lines. A recent study provided another NO- and H_2_S-releasing NSAID, NOSH-sulindac (AVT-18A), which conjugated a NO-releasing group and H_2_S-releasing group into the parent compound sulindac. NOSH-sulindac exerts a 1000- to 9000-fold growth inhibition greater than that of sulindac in human CRC cell lines HT-29, SW-480, and HCT-15. It induced apoptosis and blockage of the G_2_/M cell cycle. Preclinical data highlighted its anti-growth events against various human cancer cells without GI toxicity [99].

### 6.4. Nitric Oxide Donor Doxorubicin (NO-DOXOs)

A study conducted by Chegaev et al. designed new DOXO semisynthetic derivatives, namely NO-DOXOs, through conjugating doxorubicin with NO donor nitrooxy and phenylsulfonyl furoxan moieties. Subsequent experiments indicated that NO-DOXOs could accumulate and induce a high cytotoxicity in doxorubicin-resistant human colon cancer cells (HT29-dx) [100]. The work provided NO-releasing hybrids as a feasible strategy to address multidrug resistance (MDR) for CRC treatment.

## 7. Application and Delivery of Nitric Oxide for Colon Cancer

Nowadays, there are two foci in studies of NO in drug delivery systems for cancers: (1) augmenting the enhanced permeability and retention (EPR) effect for drug delivery; and (2) facilitating the delivery of NO to tumor sites. In this review, we focus more on the application of NO delivery in colon cancer.

### 7.1. Augmented EPR Effects by the NO Drug Delivery System

**Figure 1:**
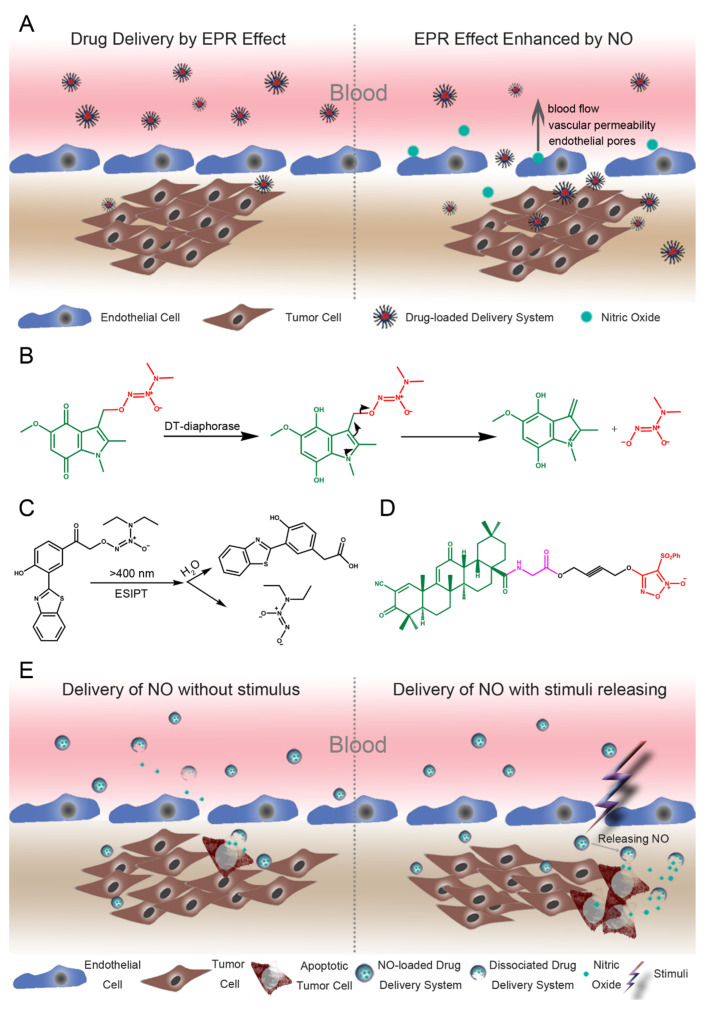
(**A**) Schematic illustration of the enhanced permeability and retention (EPR) effect by donated nitric oxide (NO). The donated NO can cause the endothelium-dependent relaxation of vascular smooth muscle, which will increase the blood flow and vascular permeability. Under this situation, the endothelial pores which serve as the gateway will be enlarged for particles and drugs; (**B**) Design of a DT-diaphorase-activated NO-donating agent; (**C**) Design of a photo-activated NO-donating agent; (**D**) Design of a transporter-mediated NO-donating agent; (**E**) Schematic illustration of nano-scaled NO delivery system with or without stimuli. Without stimulus, the NO-loaded delivery system will spontaneously release the NO in the normal tissue or tumor. For the stimuli-responsive delivery system, the release of NO can be controlled specifically by applying stimuli (e.g., ultrasound and light) at the tumor sites.

In 1986, Yasuhiro Matsumura and Hiroshi Maeda originally proposed the concept of the EPR effect, by which particles or macromolecules of a certain size tend to accumulate in the tumor tissue [129]. Typically, the newly formed tumor has unique pathophysiological characteristics, such as extensive angiogenesis and hypervasculature, defective vascular architecture, and a greatly increased production of a number of permeability mediators. Furthermore, tumor tissues usually lack effective lymphatic drainage [130]. However, due to the heterogeneity of the EPR effect, in some cases, it is difficult to deliver drugs to tumors [131]. Therefore, researchers developed various methods to augment the EPR effect via modulating many factors, such as bradykinin, nitric oxide, prostaglandins, etc. [132,133,134,135,136]. NO, as a major endothelium-dependent vasodilator produced by large blood vessels, can regulate microvascular blood flow and vascular permeability [137]. So far, many preclinical studies have demonstrated that NO can potentiate the EPR effect (Figure 1A). Clinically, an NO donor, glyceryl trinitrate, has been used to enhance the therapeutic efficacy [36].

However, since the vascular networking pattern will influence the tumor blood flow response to the systemic vasodilators, the systemic administration of a non-selective NO-donating substance may not increase—and may even reduce—the tumor blood flow. Waliul Islam et al. [138] exploited the benefit of three NO-donating agents nitroglycerin (NG), hydroxyurea, and L-arginine for the efficacy of nanomedicine by enhancing the EPR effect. After quantifying the amount of NO in tissues at 4 and 24 h after administration, they found that all three NO-donating agents could selectively increase the amount of NO in tumors. Thereafter, the administration of NO-donating agents could significantly increase (1.5–2 times) the accumulation of polymer-conjugated pirarubicin (P-THP) in the tumor. In the colon cancer C26 model, the combinational use of 5 mg/kg P-THP and NO-donating agents exerted a similar or better therapeutic effect than that of 15 mg/kg P-THP only. Moreover, compared to the P-THP alone, in the AOM/DSS-induced autochthonous murine colon cancer model, P-THP combined with NO-donating agents notably reduced both the number and size of polyps in the colon. In another study, Jun Fang et al. [139] used NG to enhance the EPR effect for the delivery of bacteria. After NG treatment, they found that the number of bacteria in tumor tissue had increased around 70-fold, 20-fold, and 10-fold at 1, 6, and 24 h, respectively. In addition, combined with NG, the bacteria could remarkedly prolong the survival length of rats with colon tumors.

### 7.2. Delivery of NO for Targeted Cancer Therapy

NO-donating agents such as S-nitrosothiols, diazeniumdiolates, and furoxans have been reported to show anti-neoplasia effects in certain cancer cells [140]. However, due to the reactive and unstable nature of these kinds of NO-donating agents, their systemic administration may cause unexpected effects resulting from non-specific activation and targeting. To precisely deliver NO to tumor sites, small molecules, and nano-scaled molecules or particles which utilize the abnormalities of the tumor microenvironment have been arousing people’s interest.

For small molecules, the targeted release of NO can be achieved by conjugating NO-donating agents to a triggering group, which can respond to the low pH situation or specifically overexpressed enzymes in the tumor tissue. The newly formed molecules usually exist as an inactive form with less systemic toxicity. An indolequinone-diazeniumdiolate was reported to exhibit the targeting ability once activated by NAD(P)H: quinone oxidoreductase 1 (DT-diaphorase), a bioreductive enzyme that is overexpressed in colon cancer [141]. Induced by DT-diaphorase, the 4,7-dioxoindole indolequinone-diazeniumdiolate was transformed into a 4,7-dihydroxyindole intermediate to liberate the lone pair electron for releasing a diazeniumdiolate anion (Figure 1B). Thereafter, at a physiological pH, the diazeniumdiolate anion will dissociate to generate NO. Another example of diazeniumdiolate employed UV or visible light to selectively release NO in the tumor tissue [142]. In this study, the photochemical NO-donating agent—O^2^-(3-(benzothiazole-2-yl)-4-hydroxyphenacyl) diazeniumdiolate—could be triggered by visible light and then release NO (Figure 1C). A more potent antitumor activity against the colon cancer cells was observed under visible light than in the dark.

In addition to the enzyme and photo triggering strategy, Yong Ai et al. [101] employed the peptide transporter 1, involved in the transportation of amino acids and peptides, to transport the NO-donating agents to the cancer cells. The authors synthesized CDDO-amino acid-NO donor trihybrids by using the amino acid to link 2-cyano-3,12-dioxooleana-1,9(11)-dien-28-oic acid (CDDO), which is an oleanolic acid derivative with antitumor activity, with the NO-donating agent furoxan (Figure 1D). The CDDO-amino acid-NO donor had more potent antitumor activity against drug-sensitive and drug-resistant colon cancer cells when compared with the compound individually. Moreover, the antitumor effect of this trihybrid has been demonstrated in colon tumor-bearing mice.

Regarding the use of nano-scaled medicines to deliver NO, NO-loaded particles utilize the EPR effect to passively target tumor tissue, followed by releasing NO spontaneously or via endogenous or exogenous stimuli (Figure 1E). The furoxan is not only developed into a tumor-directed NO-donating small molecule, but is also embedded in an amphiphilic block copolymer to form nano-scaled micelles [143]. The furoxan-bearing micelles can respond to the cysteine, which is rich in the cell, to release the NO and exert an anti-proliferative effect in colon cancer cells.

The human serum albumin (HSA), as a long-acting and safe NO delivery system, has been well-studied. Initially, HSA was used to carry NO by Stamler et al. in 1992 [144]. Although, since then, the nitrosated HSA (NO-HSA) has been investigated for various therapeutic applications, the first study on the effect of NO-HSA on cancer started in 2008 [145]. Masaki Otagiri and Toru Maruyama’s team found that NO-HSA could inhibit the proliferation of colon cancer cells in a concentration-dependent manner. Furthermore, the NO-HSA could induce the apoptosis of cancer cells to significantly suppress the tumor growth of colon tumor-bearing mice. However, NO-HSA might not exert its greatest potential because of the poor stability, insufficient circulation time, and suboptimal particle size. To overcome these drawbacks, PEGylation or a resizing strategy has been applied to modulate the NO-releasing rate and targeting capability of NO-HAS [146]. As a result, PEGylated NO-HAS became more stable both in vitro and in vivo, and the dimeric NO-HAS showed more potent antitumor activity against colon cancer. From the results of the in vivo antitumor study, they found that the antitumor activity of the NO-HSA dimer was at least 10 times higher than that of NO-HSA. Sequentially, Masaki Otagiri and Toru Maruyama’s team studied the effects of NO-HSA on the EPR effect for nano-scaled drug delivery systems, such as micelles, liposomes, and albumin nanoparticles [147,148]. They stated that the improved antitumor effect of the combinative administration of the NO-HSA dimer and nanomedicines should be attributed to the enhancement of the EPR effect by the NO-HSA. However, we found that the administration of NO-HSA alone could significantly suppress tumor growth in the results of in vivo antitumor studies. Therefore, the improved antitumor effect of combinative administration may result from the additive or synergistic effects of the NO-HSA dimer and nanomedicines.

More recently, endogenous or exogenous stimuli—such as a low pH, infrared light, and ultrasound—have been used to control NO release after NO-loaded drug delivery systems accumulate in the tumor tissue via the EPR effect. However, compared to the conventional drug delivery system, the increased complexity may limit its clinical application, although the stimuli–response drug delivery system can improve the targeting efficiency. The potential of the stimuli–response drug delivery system for delivering NO has been explored in other cancer models, and has recently been reviewed in detail [149,150]. However, few pieces of literature were found reporting the implementation of the stimuli-response drug delivery system for the delivery of NO for CRC treatment.

## 8. Clinical Manifestation of NO/NOS in Colon Cancer

Given the aggressive involvement of NOSs in many signaling pathways related to CRC initiation, NOSs have been considered to be indicators for patients with poor-prognostic colon carcinoma subtypes. Schirripa et al. investigated the feasibility of iNOS as a prognostic marker in mCRC patients; however, they failed to replicate the results [151].

Meanwhile, the efficacy of NOS chemo-inhibitors produced from plants/fruit extracts or daily nutraceuticals targeting specific NOS has been determined in preclinical work. However, few inhibitors have been advanced to clinical investigations. In patients, AtRA successfully exerts a preventive effect on UC and CAC by targeting iNOS [70].

Clinical trials have been conducted to study a commonly used NO donor, glyceryl trinitrate (GTN), which is a drug that was initially used for heart failure in clinics. A phase I trial found that GTN exerted beneficial effects on patients with colon, lung, and prostate cancers [152]. Another phase II trial suggested that GTN could improve the sensibility of patients with advanced lung cancer to chemotherapies [153].

A preclinical study found NO-donating aspirin, acetylsalicyclic acid (NCX-4016), provides protection against aspirin-induced gastric damage. In 2020, a 10-year follow-up study concluded that taking 600 mg aspirin for at least 2 years significantly reduces the incidence of colon cancer in people who are carriers of Lynch syndrome [154]. These findings suggest that NO-donating aspirin might be a good candidate for CRC therapy. A phase I trial studied the best doses and side effects of NCX-4016 in protecting patients at a high risk of CRC [151,155,156]. However, the clinical study of NCX-4016 did not move forward to Phase II and III trials for unknown reasons. Additionally, NO donors achieveed unfavorable pharmacokinetics in clinical practice[157]. Continuing efforts are still needed to prove the possibility of positioning NOS inhibitors and NO donors in the clinical arena.

## 9. Discussion and Conclusions

Conventional chemo-therapy generates poor patient outcomes due to the heterogeneity of colon cancer, resulting in chemo-/radio-resistance and MDR [158]. In recent decades, numerous attempts have been made to develop targeted strategies for CRC therapy with clinical benefits. Two decades ago, people started to study the anti-tumor effect of NO-donating agents on colon cancer [159], and proposed NO donor-based therapy as a promising strategy to CRC for clinical purposes. However, the paradoxical properties of NO are troublesome when manipulating NO as a potential therapeutic option in different microenvironments, as it may inevitably be associated with both benefits and risks.

NO donor-based therapy mainly includes NO donor single therapy; its combination with anticancer drug(s)/agent(s); and its hybridization with gaseous transmitters, such as CO or H_2_S. However, the adverse effects of traditional NO donors limit their clinical use, predominantly due to the off-target effects. Moreover, the uncontrolled release of NO has the risk of the rapid bursting of toxic agents, such as nitrates, which always results in acute toxicity in patients. To address the clinical limitations, a targeted/controlled delivery system—such as NO-donating nanoparticles—is urgently needed to specifically deliver precise doses at tumor sites to avoid cytotoxic levels in adjacent normal tissues. An imaging system for NO-donating compounds also needs to be developed to monitor and/or capture the compounds in vivo. For example, NO-donating compounds could be coupled with a fluorescent group. Most importantly, the real beneficial effects of newly developed NO donors and delivery systems need to be investigated in animal and clinical trials.

A monoclonal antibody drug is considered to be a novel NO-donating candidate with a high targeting ability and affinity. Recently, a novel antibody–drug conjugate (ADC)-like immunoconjugate—namely, antibody–nitric oxide conjugate (ANC)—drew attention as it could specifically deliver cytotoxins to tumor cells/tissues, giving an emerging missile with higher immunotherapeutic outcomes, but lower toxic effects. The developed anti-CD24 antibody G7mAb, acting as a vehicle, selectively targeted hepatic carcinoma (HCC) in vitro and in vivo. This first generation of ANC was created via coupling the NO donor HL-2 and antibody targeting CD24. This novel ANC was named HN-01. HN-01 employed an antibody to accurately deliver NO to tumor sites to achieve a superior targeted affinity and therapeutic efficacy compared to NO donors alone. However, the anticancer effect of this ANC has only been determined in liver cancer. Further investigations are needed in other types of cancers [159].

The clinical benefits of NO-targeting therapies remain obscure, partially because of the insufficient clinical data. In fact, as a widely studied area for cancer therapy, there are only a limited number of clinical trials in cancers. This might be due to multiple reasons. For example, the biphasic role of NO complicates the regimen of the therapy, which makes it hard to practice in clinical settings. In addition, other novel therapies, such as immunotherapy and gene therapy, are thriving, and have shown well-established benefits and mechanisms. This further dampens people’s interest in the seemingly ‘problematic’ NO-targeting therapies. To date, there has been only one completed phase I trial of NO-related therapy in CRC. This might be due to the successful development of the oral administration of chemotherapeutics for CRC therapy. In addition, few studies have examined the antitumor efficacy of NO delivery/controlled-releasing systems in CRC.

In addition, the clinical application of iNOS inhibitors for cancer therapy is controversial. Some studies have reported that the low level of iNOS is associated with a poor prognosis in CRC patients [160]; however, other studies have indicated a correlation between the low survival rates of patients and high level of iNOS in tumor tissues [54,161]. One study has even demonstrated that iNOS expression in tumors does not always correspond to the NO production level [162]. A clinical Phase Ib/II trial of the combinational use of NOS inhibitor L-NMMA and taxane chemotherapies is recruiting in 2020, aiming to treat refractory locally advanced or metastatic triple negative breast cancer. This trial will provide important information for the clinical application of the co-administration of NOS inhibitors with chemotherapies in cancer patients. In recent years, many natural extracts have been developed as NOS inhibitors. Although potent anticancer effects were observed in preclinical settings, none of these natural extracts have been tested in clinical trials in cancer patients. These natural products hold unique values. For example, Maqui berry itself is an edible berry, suggesting that natural extracts from it are generally considered safe. Multiple clinical trials have been performed for testing the health benefits of Maqui berry extracts. Although these trials were not conducted for cancer patients, the safety profile and PK/PD profile will be valuable for future cancer trials. However, because of the complexity of the components of these natural extracts, they are more likely to be investigated as supplements instead of cancer therapy. In addition, the beneficial effects of these natural extracts are usually achieved by targeting multiple molecules or pathways, but not specifically targeting the NO pathway.

In summary, although NO/NOS targeting therapies and NO-related drug delivery systems have shown promising results in preclinical studies, well-designed clinical studies are needed to elucidate the feasibility of this strategy in patients with CRC. There is also still an urgent need to uncover the hidden mechanisms of NO/NOS in CRC progression and management to facilitate the future development of more potent and less toxic therapies.

## Figures and Tables

**Table 1:** Paradigms of NO synthases (NOS) chemo-inhibitors for colon cancer treatment

Compound	Target	Mechanism	Inhibited Proteins	Related CRD Hallmark	Inhibited Cells	In Vivo Model	Combined Therapy	Ref.
1400 W	iNOS	Angiogenesis pathway	PROK2; MMP2	Suppress CRC cell proliferation and migration	HT29/ HCT116	Human CRC xenografts mice model	5-FU	[64,65]
L-NIO	eNOS nNOS	Angiogenesis pathway	Serpin B5; uPA	Suppress CRC cell proliferation and migration	HCT116		5-FU	[64]
Celastrol	iNOS/ eNOS	Angiogenesis pathway	IL1b, MMP-9, PDGF, TIMP-4	Suppress CRC cell proliferation and migration	HT29/HCT116		5-FU/ Salinomycin; 1400W, L-NIO	[66]
Maqui berry extracts	iNOS	Antioxidant activity; anti-inflammation	NF-κB; COX-2	Suppress CRC cell growth	HT29/ Caco-2			[67]
*M. alba* extract	iNOS	Caspase 3 activity		Induce apoptosis in human CRC cells	HCT-15			[68]
PBISe	iNOS/Akt	MAP and PI3 kinase signaling	pAkt; Akt2	Induce apoptosis in human CRC cells	Caco-2			[68]
Nobiletin (NOB)	iNOS	Nrf2 signaling pathway; antioxidative activity	cyclin D, CDK6, CDK4, CDK2	Suppress colitis-associated colon carcinogenesis; caused cell cycle arrest in human CRC cells	RAW 264.7/ HCT116	AOM/DSS-treated mice		[69]
All-Trans Retinoic Acid (AtRA)	iNOS/ TNF-α	TLR4/NF-κ;B signaling pathway		Improve the clinical prevention of CAC		colonic mucosa of patients with CAC and UC		[70]
Fucoxanthin-Rich Brown Algae Extract (FX-BAE)	iNOS	Pro-inflammatory cytokines; TNF-α and IL-6		Reduced intestinal injury caused by chronic inflammation; attenuated colitis-associated colon cancer		DSS-induced colitis and CACC in mice		[71,72]
DHA	iNOS	Cyclin-dependent kinase		Induce apoptosis in human CRC cells	Caco-2			[73,74]
Polyphenol Ellagic Acid	iNOS/COX-2/TNF-α/IL-6	NF-κB pathway	COX-2; NF-κB; iNOS	Attenuated colonic inflammation		Inflammatory rat model		[75]
Phaleria macrocarpa Stem Bark	iNOS			Inhibit iNOS expression	HCT116			[76]
Cannabidiol (CBD)	eNOS	Autophagy; antioxidant activity	phospho-eNOS; SOD2	Overcome oxaliplatin resistance	DLD-1/colo205	human CRC xenografts mice model	Oxaliplatin and other chemotherapeutics	[77]
Cetuximab	iNOS	Immunosuppressive activation	IL-10; TGFβ	Potentiate chemotherapeutic efficacy		Patients with CRC or metastatic CRC	Chemotherapeutics	[78]

**Table 2:** Paradigms of NO donors for colon cancer treatment

NO Donor	Properties	Mechanism	Chemo-Preventative Effects	Inhibited Cells	In Vivo Model	Ref.
NO-NSAIDs			Suppress CRC cell proliferation; block cell cycle transition	HT-29		[95]
NO-aspirin (NO-ASA)	NO-NSAID	Induce COX-2; Inhibit the β-catenin/TCF4 signaling pathway	Inhibit CRC cell growth	HT-29/DLD-1		[95]
NCX-4016	NO aspirin derivate	Independent of any inhibitory activity on COX-1 or COX-2	Reduce aberrant crypt foci (ACF) in colon		Azoxymethane (AOM)-induced mice	[97]
NOSH–aspirin (NBS-1120)	NO- and H_2_S-releasing NSAID	Inhibit cyclo-oxygenase enzyme activity	Suppress CRC cell proliferation; induce apoptosis; block cell cycle	HT-29	Human xenograft mouse model	[98]
NOSH-sulindac (AVT-18A)	NO- and H_2_S-releasing NSAID	Inhibit COX-1 and COX-2	Anti-colon cancer activity and anti-inflammation	HT-29/SW-480/HCT-15	Rat	[99]
NO donor Doxorubicin (NO-DOXOs)		Inhibit cellular drug efflux; nitration of tyrosine residues of MRP3 protein	Induce cytotoxicity in colon cancer cells;	doxorubicin-resistant HT-29		[100]
CDDO-Amino Acid-Nitric Oxide Donor Trihybrids		Inhibit HIF-1α, ERK, Stat 3, and AKT signaling	Anti-tumor effect against chemo-sensitive and chemo-resistant CRC	HCT-8; HCT-8/5-FU		[101]
Glyceryl trinitrate (GTN)		Activate caspase-1 and caspase-10	Induce apoptosis in colon cancer cells	HCT116	In clinics	[102]
GT-094	NO-NASID	Activate the ROS-miR-27a:ZBTB10-Sp transcription factor pathway	Inhibit CRC cell growth	RKO/SW480		[103]

**Table 3:** In vivo paradigms of NOS chemo-inhibitors/NO donors for colon cancer treatment

Compound	In Vivo Model	Mechanism	Treatment (Dose/Duration)	Therapeutic Efficacy	Ref.
1400W	Genetically engineered xenograft mice model with constitutive iNOS expression (colon adenocarcinoma DLD-1)	Angiogenesis pathway	6 mg/kg-1/h-1 1400W/13 days	Inhibited tumor growth	[65]
Nobiletin (NOB)	AOM/DSS colon cancer model on CD-1 mice	iNOS ↓ antioxidative enzymes ↑ cell cycle ↓	AIN93G diet containing NOB (0.05 wt % in diet)/ 1 week after the AOM injection until the end of study	Suppressed colitis-associated colon carcinogenesis	[69]
All-Trans Retinoic Acid (AtRA)	Ex vivo; colonic mucosa of patients with CAC and UC	LPS/TLR4/NF-κB signaling pathway NOS2 ↓ TNF-α ↓	10–7 M AtRA/6 h; stimulated with 10 μg/mL lipopolysaccharide (LPS)	Clinically prevented the CAC development and progression	[70]
Fucoxanthin-Rich Brown Algae Extract (FX-BAE)	DSS-induced colitis and CACC model in BALB/c mice	Oxidative stress↓	Colitis: fed with FX-BAE 1, 2, or 5 g/kg/day from day 8 to day 14; CACC: fed with FX-BAE at 0.5, 1, or 2.5 g/kg every 2 days	Decreased the incidence of colonic neoplasm; increased superoxide dismutase (SOD) production, lymphocyte proliferation; prolonged survival rate in CACC mice	[71,72]
Polyphenol Ellagic Acid	1,2-dimethylhydrazine-induced colon cancer model on Wistar albino rats	Anti-inflammatory NF-κB pathway ↓ iNOS ↓ COX-2 ↓ TNF-α ↓ IL-6 ↓	60 mg/kg ellagic acid/p.o./every day for 15 weeks	Chemo-prevention on colon carcinogenesis	[75]
Cannabidiol (CBD)	Colo205 xenograft model on BALB/c nude mice	CBD overcomes NOS-induced oxaliplatin resistance by inducing autophagy	CBD + oxaliplatin (i.p.)	Overcame the resistance to oxaliplatin	[77]
Cetuximab	Ex vivo; CRC tissue explant culture	iNOS ↓ immunosuppressive cytokines ↑	Cetuximab + chemotherapy (CTX + Chemo)	Potentiated the chemo-therapeutic efficacy	[78]
NCX-4016	TNBS-AMO colon cancer model on rats	Independent of any inhibitory activity on COX-1 or COX-2	10 mg/kg NCX-4016 after four- weeks administration of AOM	Reduce aberrant crypt foci (ACF) in colon	[97]
NOSH–aspirin (NBS-1120)	Human colon cancer xenograft model	cell proliferation ↓ apoptosis ↑ the blockage of G(0)/G(1) cell cycle		Reduced the tumor volume by 85%	[98]
